# Association between Blood Lead Levels and Silent Myocardial Infarction in the General Population

**DOI:** 10.3390/jcm13061582

**Published:** 2024-03-10

**Authors:** Mohamed A. Mostafa, Mohammed A. Abueissa, Mai Z. Soliman, Muhammad Imtiaz Ahmad, Elsayed Z. Soliman

**Affiliations:** 1 Epidemiological Cardiology Research Center (EPICARE), Department of Internal Medicine, Section Cardiology, Wake Forest School of Medicine, Winston-Salem, NC 27157, USA; mmostafa@wakehealth.edu; 2 Department of Cardiothoracic Surgery, Al Manial Specialized Cairo University Hospital, Cairo 11956, Egypt; mohammedabuessa1991@hotmail.com; 3 Wake Forest University, Winston-Salem, NC 27106, USA; maisoliman2003@outlook.com; 4 Department of Internal Medicine, Section on Hospital Medicine, Medical College of Wisconsin, Wauwatosa, WI 53226, USA; imtiaz186@gmail.com

**Keywords:** silent myocardial infarction, lead exposure, cardiovascular disease, NHANES-III

## Abstract

**Background**: Although the link between lead exposure and patterns of cardiovascular disease (CVD) has been reported, its association with silent myocardial infarction (SMI) remains unexplored. We aimed to assess the association between blood lead levels (BLLs) and SMI risk. **Methods**: We included 7283 (mean age 56.1 ± 2.52 years, 52.5% women) participants free of CVD from the Third National Health and Nutrition Examination Survey. BLL was measured using graphite-furnace atomic absorption spectrophotometry. SMI was defined as ECG evidence of myocardial infarction (MI) without history of MI. The association between SMI and BLLs was examined using multivariable logistic regression. **Results**: SMI was detected in 120 participants with an unweighted prevalence of 1.65%. Higher BLL correlated with higher SMI prevalence across BLL tertiles. In multivariable-adjusted models, participants in the third BLL tertile had more than double the odds of SMI (OR: 3.42, 95%CI: 1.76–6.63) compared to the first tertile. Each 1 µg/dL increase in BLL was linked to a 9% increase in SMI risk. This association was consistent across age, sex, and race subgroups. **Conclusions**: Higher BLLs are associated with higher odds of SMI in the general population. These results underscore the significance of the ongoing efforts to mitigate lead exposure and implement screening strategies for SMI in high-risk populations.

## 1. Introduction

Lead exposure is estimated to account for nearly 1 million deaths globally in 2019, with 850,000 deaths attributed to its cardiovascular effects [1]. Long-term lead exposure contributes to 4.6% of the global burden of CVD [2]. Higher levels of heavy metal exposure including lead have been linked to coronary heart disease (CHD), high blood pressure, heart failure, cardiovascular, and all-cause mortality [3,4,5,6]. Beyond its cardiovascular effects, lead exposure has a wide-ranging impact on multiple bodily systems, including the neurological, hepatic, and renal systems [7,8]. Although blood lead levels (BLLs) have been decreasing in the past decade, almost half of the US population is at risk of adverse health outcomes caused by high lead levels in their early childhood [9,10]. Human exposure to lead nowadays mainly occurs through food, water, tobacco smoke, and e-cigarettes, either by inhalation, ingestion, or skin contact [11,12]. According to the US Centers for Disease Control and Prevention (CDC), there is no safe level of lead exposure for adults, as adverse health effects are anticipated at all exposure levels [13]. Even exposure concentrations as low as 2 µg/dL are considered to pose a cardiovascular hazard [14].

Silent myocardial infarction (SMI) imposes a significant health burden due to its absence of symptoms and the lack of established screening protocols. While prior research has established the adverse effects of lead exposure on cardiovascular health and mortality, the potential link between lead exposure and SMI remains unexplored [15,16]. It is believed that the atherogenic effects of lead exposure are attributed to the generation of excessive free radicals, causing oxidative stress and endothelial injury that impair endothelial growth and repair processes [17,18]. Considering the non-specific symptoms of SMI and the global public health impact of lead exposure, it is crucial to identify effective measures for early detection, screening, and strategies to mitigate the overall burden of lead exposure. This study aims to investigate the possible association between blood lead levels and SMI in the general population, emphasizing the public health implications of this relationship.

## 2. Materials and Methods

### 2.1. Study Population

This study utilized data from the Third National Health and Nutrition Examination Survey (NHANES-III), which was conducted from 1988 to 1994. NHANES-III is part of a series of health surveys aimed at evaluating the health and nutritional status of the non-institutionalized United States population. Participants completed a household interview, laboratory measurements, and physical examinations. During the survey, participants underwent household interviews, physical examinations, and laboratory measurements. Comprehensive information on the survey design, protocol, response rates, and specific data collection methods has been previously published [19].

A written informed consent was obtained from all participants prior to data collection. NHANES III was approved by the National Center for Health Statistics (NCHS) Research Ethics Review Board (ERB).

For this analysis, we only considered NHANES-III participants who underwent ECG recording (*n* = 8561). We excluded participants with missing key covariates or with ECG conditions that prohibit the appropriate detection of MI by the Minnesota ECG Classification [15]. This includes the presence of a complete left bundle branch block, pre-excitation, or ventricular pacemaker. To eliminate confounding factors and identify SMI cases, we excluded those with a history of cardiovascular disease, including history of clinical MI. After all exclusions (*n* = 1278), 7283 participants remained and were included in the analysis.

### 2.2. Assessment of Covariates

Demographic information regarding age (continuous in years), sex (male and female), race/ethnicity (white, black, and other), and smoking status (current smokers: smoked ≥100 cigarettes in their lifetime and were currently smoking) were ascertained based on responses to standardized questionnaires administered during an in-home interview. Hypertension was defined as systolic blood pressure (BP) ≥130 mmHg or diastolic BP ≥ 85 mmHg or the use of antihypertensive medications. Obesity was defined as body mass index (BMI) ≥ 30 kg/m^2^. Diabetes was defined as a fasting plasma glucose ≥ 126 mg/dL, hemoglobin A1c values ≥ 6.5%, or previous use of diabetes-related medications. Total cholesterol was assessed from blood sample collected by venipuncture during the physical examination.

### 2.3. Ascertainment of SMI

During a physical examination conducted in a mobile examination unit, resting digital 12-lead electrocardiograms (ECGs) were obtained using a Marquette MAC 12 electrocardiograph (Marquette Medical Systems, Milwaukee, WI, USA). Subsequently, the ECGs underwent visual inspection by trained technicians, followed by automated processing of the tracings at the Epidemiological Cardiology Research Center (EPICARE Center, Wake Forest School of Medicine, Winston-Salem, NC, USA). ECG abnormalities, including MI, were classified using the Minnesota ECG Classification which was defined as minor Q waves plus major ST-T abnormalities in any lead (MC 1-3-X with 4-1-X, or 4-2, or 5-1, or 5-2) or major Q waves (MC 1-1-X or 1-2-X); Further description of ECG Minnesota Codes involved in the definitions of MI are provided elsewhere [20]. SMI was defined as the presence of ECG evidence of MI using the standards of the Minnesota ECG Classification in the absence of a prior history of MI.

### 2.4. Lead Exposure Ascertainment

During the physical examination conducted as part of NHANES III, whole-blood specimens were collected via venipuncture from all survey participants aged 1 year and older [19]. These specimens were then sent to the Division of Laboratory Sciences at the National Center for Environmental Health of the Centers for Disease Control and Prevention (CDC) for analysis of lead concentration. Blood lead levels (BLLs) were quantified using graphite furnace atomic absorption spectrophotometry. The limit of detection for BLLs in NHANES III is 0.07 µg/dL, with results below this limit reported as a value equal to the detection limit divided by the square root of 2 [21].

### 2.5. Statistical Analysis

Demographics and clinical characteristics of the participants were compared across tertiles of BLLs using analysis of variance (ANOVA) for continuous variables and chi-square for categorical variables. Odds ratios (ORs) and 95% confidence intervals (CIs) for the risk of SMI were estimated using logistic regression models across tertiles of BLL, with the first tertile as a reference group. Two multivariable-adjusted models were constructed: Model 1 adjusted for age, sex, race, and income levels, and Model 2 adjusted for Model 1 plus systolic blood pressure, obesity, diabetes, smoking, total cholesterol, antihypertensive medications, and lipid-lowering medications. Similar models were utilized to examine the odds of SMI associated with each 1 µg/dL increase in BLLs. We also examined the effect modification of the association between tertiles and SMI by age (<65 vs. >65 years), sex (men vs. women), and race (white vs. non-white). Interaction with the main effect was tested in a model adjusted similarly to Model 2.

All statistical analyses were performed using SAS version 9.4 (SAS Institute Inc., Cary, NC, USA), incorporating a complex sampling design (primary sampling units, sampling strata, and weights), and two-sided *p*-values of less than 0.05 were considered significant.

## 3. Results

After all exclusions *(n* = 1278), 7283 participants (mean age 56.1 ± 2.52 years, 52.5% women, 81% white) remained and were included in the analysis. The BLLs ranged from 0.70 µg/dL to 16.4 µg/dL as follows: first tertile (0.70–2.60 µg/dL), second tertile (2.70–4.60 µg/dL), third tertile (4.70–16.4 µg/dL). In our sample, 120 participants had SMI (unweighted percentage = 1.65%). Participants with higher levels of BLL tertiles had a higher prevalence of SMI (*p* < 0.001). As shown in (Table 1), participants with higher BLLs were more likely to be older, currently smoking, men, with income level <20 K annually, and with higher blood pressure levels. In a multivariable logistic regression model adjusted for socio-demographics (age, sex, race, income level) and common CVD risk factors (high blood pressure, obesity, diabetes, smoking, total cholesterol, antihypertensive medications, and lipid-lowering medications), participants with BLL levels in the third tertile had more than double the odds of SMI compared to those with BBL levels in the first tertile ((OR (95% CI): 3.42 (1.76–6.63)). Each 1 µg/dL increase in BLL was associated with 9% higher odds of SMI ((OR (95% CI): 1.09 (1.05–1.14)) (Table 2). The association between BLL tertile and SMI was consistent among participants stratified by sex (men vs. women), race (white vs. non-white), age (<65 years vs. ≥65 years) (Table 3).

## 4. Discussion

In the United States, around 170 million had BLLs above 5 µg/dL during their early life, with lacking evidence of its associated adverse health consequences [1,10]. In this analysis from a large community-based population, we showed that higher BLLs are associated with a higher prevalence of SMI. The likelihood of SMI tripled as the BLL tertiles increased. This study adds to the growing body of evidence according to which lead exposure is a risk factor for cardiovascular disease [15,16]. Populations with elevated BLLs may be at increased risk for SMI and more efforts should be directed toward proper screening and preventive interventions.

The prevalence of lead exposure peaked in the late 1970s, particularly among children aged 1–5 years with BLLs greater than or equal to 10 µg/dL [22]. While there has been a steady decline in BLLs in recent decades, socioeconomic status and racial inequalities continue to influence population exposure to environmental hazards, albeit to a lesser extent compared to the late 1970s [9,23]. At that time, African Americans had higher national BLLs compared to the white population, with a mean blood lead level of 23 µg/dL in low-income black children [24]. Moreover, certain occupations such as mining, construction, and manufacturing, particularly battery manufacturing, involve significant exposure to environmental hazards including lead [11]. Despite this progress, the long-term health consequences of lead exposure are still being investigated, and it is imperative to continue efforts to reduce exposure and minimize its adverse effects.

The asymptomatic nature of SMI and the absence of formal screening protocols significantly contribute to its health burden. Prevalence rates of SMI are influenced by age groups and socioeconomic status, and it is frequently associated with comorbidities such as hypertension, diabetes, and previous coronary heart disease (CHD) [25,26]. In the general population, SMI has been linked to an increased risk of sudden cardiac death and heart failure [27,28].

Our study reveals that individuals in the highest tertile of BLLs tended to be older men with a higher incidence of SMI. This, in conjunction with the gradual onset of CHD and the typical age group affected, may contribute to the association between elevated BLLs and the incidence of ischemic events in older participants. Furthermore, individuals in this age group may have experienced higher lead exposure during their early childhood [5]. Furthermore, lead can remain in the human bones for up to 30 years, potentially serving as a source of continuously circulating lead long after the cessation of external exposure, emphasizing the cumulative and long-term effects of lead exposure on CVD [29].

Prior research has demonstrated a significant association between environmental lead exposure and CVD, with unfavorable outcomes in cases of myocardial infarction (MI) and out of hospital cardiac arrest [30]. For instance, Afridi et al. conducted a study wherein they observed elevated levels of lead in hair samples collected from MI patients in comparison to control subjects. Importantly, the lead levels displayed a progressive increase corresponding to the severity of the disease, with the highest concentrations identified in individuals experiencing their third episode of MI [31]. Additionally, a systematic review of more than 300,000 participants revealed an 85% increased risk for the development of coronary heart disease (CHD) among individuals in the high tertile of lead exposure, underscoring lead’s significance as a potential risk factor for CHD [32].

The perception of anginal pain during myocardial ischemia involves intricate interactions between the myocardium and the nervous system [33]. Autonomic neuropathy, variations in pain thresholds, and altered neural processing in the peripheral and central nervous systems contribute to the pathophysiology of asymptomatic myocardial ischemia [34,35,36]. The adverse effects of lead exposure on both the cardiovascular and nervous systems could possibly explain these findings. First, lead exposure induces oxidative stress and chronic inflammation, leading to endothelial dysfunction and accelerated atherosclerosis [18,37]. Second, it promotes the development of atherosclerosis and plaques, as demonstrated in animal models exposed to low-level lead [38]. Furthermore, lead impairs the release of tissue plasminogen activator (t-PA) and increases plasminogen activator inhibitor-1 release, which can lead to coagulation abnormalities and a heightened risk of thrombosis, especially in the presence of endothelial injury [39]. Moreover, lead exposure-related neurological dysfunctions, such as peripheral neuropathy and cortical dysfunction, can alter the perception of anginal pain in affected individuals [40,41]. Another important consideration regarding SMI and lead exposure is the influence of socioeconomic status and education levels [42]. Populations with socioeconomic disadvantage tend to undergo higher levels of toxic metals exposure including lead, and lead exposure has been associated with cognitive decline and cortical dysfunction [43]. Adverse cognitive effects of lead exposure further impact the health literacy in this population and thus the poor comprehension of symptoms related to myocardial infarction [44].

Given the combined impact of lead exposure and the significant health and economic burden associated with SMI, effective screening and preventive measures are imperative for this high-risk population. Our findings highlight the importance of recognizing low-level environmental lead exposure as a crucial cardiovascular risk factor. Incorporating lead exposure into risk stratification strategies can aid in identifying high-risk groups and promoting targeted screening for CHD.

## 5. Conclusions

In this study, we demonstrate that higher BLLs are linked to higher odds of SMI. To ensure standardized ascertainment methods, we used ECG data to define SMI as the primary outcome, similar to prior studies. Although this may ensure reproducibility in future research, SMI cases were not further confirmed by other diagnostic modalities including echocardiography, which could be a limitation for this study. Additionally, several covariates were assessed by self-report, which is liable for recall bias and confounding remains a possibility despite adjusting for several factors. Finally, we could not establish causality or temporality given the cross-sectional nature of this study. Its key strengths include the large sample size and standardized ascertainment of variables. Our results emphasize the importance of ongoing efforts to reduce lead exposure and establish comprehensive screening protocols for SMI among high-risk groups. Future research should be target-investigating the underlying mechanisms behind this association and exploring potential racial and socioeconomic disparities. Such insights will facilitate the development of more targeted and evidence-based preventive measures which could have a favorable impact on CVD prevention.

## Figures and Tables

**Table 1:** Baseline characteristics of the study participants.

Characteristics *	Blood Lead Levels Tertiles			*p*-Value !
	1st Tertile *n* = 2369	2nd Tertile *n* = 2451	3rd Tertile *n* = 2463	
Age (years)	53.3 ± 0.45	56.8 ± 0.45	58.4 ± 0.52	<0.0001
Men	638 (30.4%)	1166 (47.0%)	1641 (64.4%)	<0.0001
White	1293 (84.0%)	1267 (81.7%)	1070 (77.7%)	<0.0001
Income level <20K	900 (23.6%)	1053 (26.9%)	1308 (38.2%)	<0.0001
Systolic Blood Pressure (mmHg)	124.8 ± 0.61	128.9 ± 0.66	131.6 ± 0.67	<0.0001
Diastolic Blood Pressure (mm Hg)	75.2 ± 0.28	76.7 ± 0.38	77.1 ± 0.36	<0.0001
Antihypertensive Medications	475 (15.8%)	551 (20.1%)	513 (17.3%)	0.01
Diabetes	315 (8.3%)	300 (8.1%)	277 (8.4%)	0.94
Current smoker	278 (12.7%)	513 (23.0%)	855 (36.1%)	<0.0001
Obesity	769 (28.3%)	708 (24.6%)	558 (22.5%)	0.009
Total cholesterol	212.5 ± 1.3	219.9 ± 1.4	219.2 ± 1.3	0.01
Lipid-lowering medications	74 (3.4%)	75(3.3%)	59(3.4%)	0.99
Silent MI	20 (0.4%)	32(0.9%)	68 (2.4%)	<0.0001

Continuous variables are presented as mean (standard error) and categorical variables as count (percentage). All percentages and means ± SE are weighted for complex survey design to be nationally representative estimates. ! *p*-value by *t*-test for continuous variable or chi-square for categorical variables.

**Table 2:** Association of blood lead levels with silent myocardial infarction.

Blood Lead Levels	Events/Participants *n* (%)	Model 1 OR (95% CI)	*p*-Value	Model 2 OR (95% CI)	*p*-Value
First Tertile (0.70–2.60 µg/dL)	20/2369 (0.4%)	Ref	-	Ref	-
Second Tertile (2.70–4.60 µg/dL)	32/2451 (0.9%)	1.51 (0.69, 3.34)	0.43	1.44 (0.64, 3.25)	0.42
Third Tertile (4.70–16.4 µg/dL)	68/2463 (2.4%)	3.73 (1.95, 7.11)	<0.0001	3.42 (1.76, 6.63)	<0.0001
Per 1 µg/dL	120/7283 (1.2%)	1.10 (1.06, 1.15)	<0.0001	1.09 (1.05, 1.14)	<0.0001

OR (95% CI) = Odds ratio (95% Confidence Interval). Model 1 adjusted for age, sex, race, income levels. Model 2 adjusted for model 1 plus systolic blood pressure, obesity, diabetes, smoking, total cholesterol, antihypertensive medications, and lipid-lowering medications.

**Table 3:** Subgroup analysis for the association between tertiles of blood lead levels and silent myocardial infarction.

Subgroups	BLL Tertiles *	Silent MI *n* (%)	OR (95% CI) †	Interaction *p*-Value
Men	2nd Tertile	15 (1.2%)	2.83 (0.52, 15.1)	0.50
	3rd Tertile	49 (2.9%)	7.68 (1.83, 32.1)	
Women	2nd Tertile	17 (1.3%)	1.05 (0.43, 2.54)	
	3rd Tertile	19 (2.3%)	2.25 (0.96, 5.29)	
White	2nd Tertile	17 (1.3%)	1.37 (0.55, 3.38)	0.40
	3rd Tertile	35 (3.2%)	3.78 (1.83, 7.83)	
Non-White	2nd Tertile	15 (1.2%)	1.91 (0.43, 8.54)	
	3rd Tertile	33 (2.3%)	1.74 (0.51, 5.90)	
<65 years	2nd Tertile	13 (0.8%)	1.29 (0.44, 3.77)	0.75
	3rd Tertile	21 (1.3%)	2.39 (0.90, 6.36)	
≥65 years	2nd Tertile	19 (2.2%)	1.86 (0.65, 5.33)	
	3rd Tertile	47 (5.1%)	5.83 (2.12, 15.9)	

* Reference group is first-tertile BLLs. † Model adjusted for age, sex, race, income levels, systolic blood pressure, obesity, diabetes, smoking, total cholesterol, antihypertensive medications, and lipid-lowering medications.

## Data Availability

Data used in this study are publicly available at: https://wwwn.cdc.gov/nchs/nhanes/nhanes3/datafiles.aspx. Accessed on 1 March 2023.
